# Supplementary figures and images for: POMC neurons control fertility through differential signaling of MC4R in kisspeptin neurons
Source: eLife. 2025 Jul 17;13:RP100722. doi: 10.7554/eLife.100722 (PMC12270483; doi:10.7554/eLife.100722)

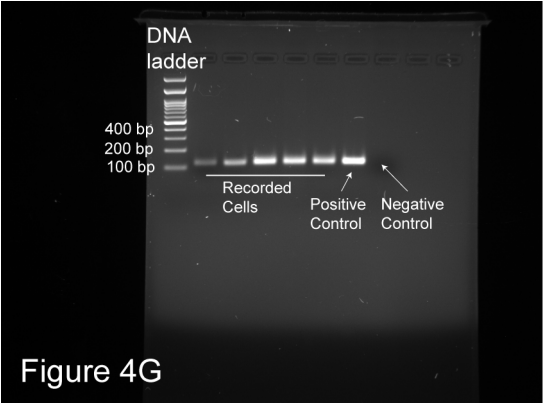

Supplement: Figure 4—source data 1. [file elife-100722-fig4-data1.pdf]

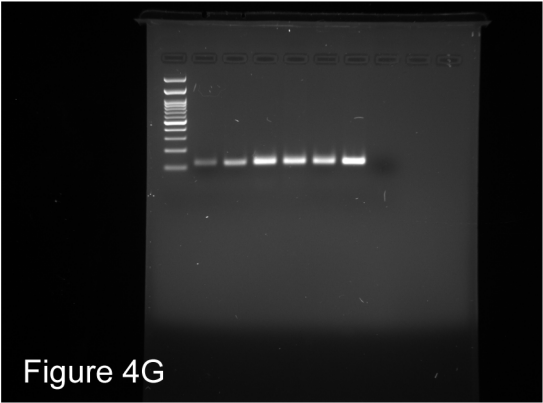

Figure 4G

Supplement: Figure 4—source data 2. [file elife-100722-fig4-data2.pdf]
